# A systematic review on the implication of *Candida* in peri-implantitis

**DOI:** 10.1186/s40729-021-00338-7

**Published:** 2021-06-17

**Authors:** Irene Lafuente-Ibáñez de Mendoza, Amaia Cayero-Garay, Guillermo Quindós-Andrés, José Manuel Aguirre-Urizar

**Affiliations:** 1 Department of Stomatology II, University of the Basque Country (UPV/EHU), Barrio Sarriena s/n, 48940 Leioa, Vizcaya Spain; 2 Department of Immunology, Microbiology and Parasitology, University of the Basque Country (UPV/EHU), Leioa, Vizcaya Spain

**Keywords:** *Candida*, Peri-implantitis, Systematic review

## Abstract

**Background:**

*Candida* is a heterogeneous fungal genus. Subgingival sulcus is a refuge for *Candida*, which has already been related to the pathogenic inflammation of periodontitis. This work aims to review the presence of *Candida* in the sulcular fluid surrounding dental implants and discuss its potential role in peri-implantitis.

**Results:**

A bibliographical research was performed in PubMed, Scopus and Web of Science databases, with the keywords *candida*, peri-implantitis, periimplantitis, “dental implant” and implant. Newcastle-Ottawa Scale was used to assess the methodological quality of the included studies. At the end, nine observational studies were included, which analysed 400 dental implants with PI and 337 without peri-implantitis. Presence of *Candida* was assessed by traditional microbiological culture in blood agar or/and CHROMagar, though identification was also detected by quantitative real-time PCR, random amplified polymorphic DNA or ATB ID 32C. Dentate individuals and implants with peri-implantitis (range, 3–76.7%) had a bigger presence of *Candida*. *C*. *albicans* was the most isolated species, followed by *Candida parapsilosis*, *Candida tropicalis*, and *Candida dubliniensis*.

**Conclusion:**

*Candida* is part of the microbiological profile of the peri-implant sulcular fluid. More studies are needed to compare the link between *Candida* and other microorganisms and to discover the true role of these fungi in peri-implantitis.

## Background

Oral rehabilitation with dental implants is a predictable and safe therapeutic procedure to treat tooth loss, in both partially and completely edentulous patients. Throughout the years, more advances have been made in the design, surface and chirurgical protocols of the implant systems, whose success can reach up to 95% of cases [1, 2].

Mechanical and biological complications are the main cause of dental implant failure. Peri-implantitis (PI) is a multifactorial infectious disease characterized by inflammation in the peri-implant mucosa and a progressive loss of supporting bone [3]. Although inflammatory response is more pronounced in the tissues surrounding the implants than in those surrounding the teeth [4], the microbiological environment associated to PI is similar to the observed in conventional periodontal disease, which includes anaerobic Gram-negative bacteria, such as *Prevotella nigrescens*, *Campylobacter rectus* and *Aggregatibacter actinomycetemcomitans* [5]. Other microorganisms, for example fungi, like *Candida*, could also participate in the onset and development of PI, since *Candida* colonization and biofilm formation is relatively common on other metallic surfaces, like hip and knee prostheses [6, 7]. However, there is a direct association between surface roughness and hydrophobicity with biofilm development as surface topography influenced microbial adhesion. For instance, titanium is one of the biomaterials most resistant to microbial colonization and to the development of *C*. *albicans* and bacterial biofilms [8].

*Candida* is a commensal of the oral cavity that can be isolated from many healthy individuals but can trigger mucosa infections (candidiasis) associated to different predisposing factors, like immunodeficiency [9]. Subgingival sulcus can also play as a refuge for pathogenic fungi [10]. In addition, different species of *Candida* have already been associated with the maintenance of periodontal inflammation in periodontitis [11, 12]. Nevertheless, the mechanisms by which *Candida* may enhance bone resorption, especially in the jaws, are unclear.

*Candida albicans* is the species more frequently isolated in PI, developing thick biofilms over the peri-implant surface [10]. Given its ability to adhere to the implant area in intimate contact with the bone, it has been hypothesized that *Candida* could also contribute to the progression of PI, but this link has yet to be elucidated.

The objective of this work is to make a systematic review of the literature, aiming to recognize the presence of different species of *Candida* in the peri-implant niche and to discuss its role in the pathogenesis and progression of peri-implantitis.

## Methods

### Research strategy

The methodological design of this study matches the PRISMA criteria and guidelines [13]. In this systematic review, we address the question “what is the role of *Candida* in the development of peri-implantitis?”.

Two independent co-authors (ILIM, ACG) performed a systematic bibliographical research in PubMed (US National Gallery of Medicine), Scopus and Web of Science/Knowledge. The search strategy consisted in different combinations of the MeSH keywords: *Candida*, peri-implantitis, periimplantitis, “dental implant” and implant (*candida* AND peri-implantitis; *candida* AND periimplantitis; *candida* AND dental implant; *candida* AND implant).

### Inclusion and exclusion criteria

Inclusion criteria were studies published in English or Spanish until December 2020 in patients with dental implants and diagnosis of PI and/or analysing samples from the sulcular fluid surrounding dental implants. Among exclusion criteria were case reports, reviews, position papers, and author opinions. Moreover, those studies not available in full format and experimental studies were also excluded.

### Selection of the studies and data collection

The study selection and data extraction were also performed by two reviewers (JMAU and ILIM). A third one (JMAU) participated in the decision-making in case of doubt about the inclusion of the studies. Also, if essential data for the review was missing or unclear, the corresponding author clarified the problem.

The titles and abstracts of the retrieved references were screened for relevance and after this, the full texts of all articles potentially eligible were analysed against the inclusion/exclusion criteria. In order to collect the information of the different studies retrieved, a standard document was utilized for data regarding authors, year of publication, study design, diagnostic criteria, clinical specimens, implant systems used, and microbiological methods. In addition, quantitative data on the isolation of *Candida* and other microorganisms were collected for both peri-implantitis and healthy implants.

For categorical variables, we performed a descriptive statistical analysis to obtain frequencies and percentages, as well as to determine the average and standard deviation for quantitative variables.

### Risk of bias

A modified Newcastle-Ottawa Scale (NOS) was used to assess the methodological quality of the included studies [14]. This system analyses the risk of bias of nonrandomized studies, taking into account three domains and eight items for cohort studies: selection, comparability and outcome. The total maximum score is 9; a study with a score from 7 to 9 has high quality; 4 to 6, high risk of bias; and 0 to 3, very high risk of bias.

## Results

### Bibliographical search and retrieval

**Fig. 1:**
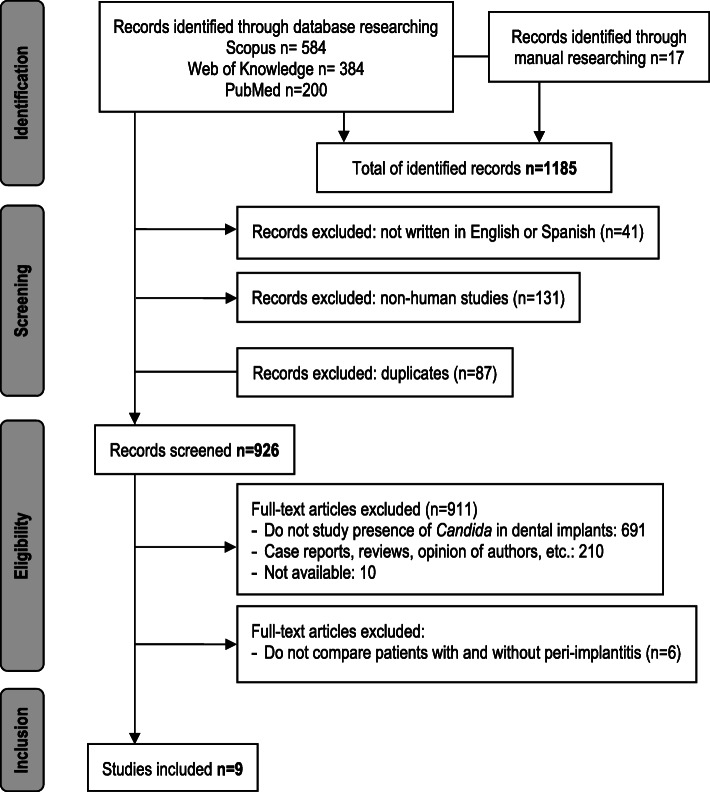
PRISMA flow diagram. Synthesis of the bibliographical analysis

The selection process of search and retrieval of literature is showed in Fig. 1. Initially, 1185 records were retrieved from databases, from which 41 were excluded due to not being published in English or Spanish, 131 because they were not made in humans and 87 for being duplicates. Thus, 926 articles were screened, but 691 were eliminated because they did not study the presence of *Candida* in dental implants, another 210 for being case reports, reviews or author opinions and other 10 for not being available in full-text.

After the detailed analysis, 15 articles studying the presence of *Candida* in patients with dental implants were included. However, during the extraction data process, we saw that only nine of them compared patients with and without PI, which accounted for the 0.78% of the initial number (Table 1) [10, 15–22]. So, the remaining six manuscripts were discarded since they studied patients with dental implants but without diagnosis of the peri-implant status [2, 23–27].

**Table 1:** Clinical data of the included studies

Authors and year	Country	Assessment of ***Candida***	Patients			Mean age (years)	Dental implants		
			Total	PI	Healthy		Total	PI	Healthy
Rosenberg et al. 1991 [15]	USA	-	75	11	64	-	83	-	-
Leonhardt et al. 1999 [16]	Sweden	Blood agar	88	37	51	63	-	-	-
Listgarten et al. 1999 [17]	USA	Blood agar	41	41	0	59	44	44	0
Albertini et al. 2015 [18]	Spain	CHROMagar	33	33	0	67.1	48	48	0
Canullo et al. 2015 [19]	Italy	qPCR	534	53	481	62,25	235	231	1276
Schwarz et al. 2015 [20]	Germany	qPCR	29	19	10	-	29	19	10
Bertone et al. 2016 [21]	Argentine	CHROMagar	40	40	0	56	89	49	40
Alrabiah et al. 2019 [10]	Saudi Arabia	CHROMagar	84	43	41	53,65	84	43	41
Alsahhaf et al. 2019 [22]	Saudi Arabia	CHROMagar	126	84	42	55,77	126	84	42

*PI* peri-implantitis

### Clinical and microbiological characteristics of the included studies

The microbiological analysis was performed, in all the studies, collecting samples from the sulcular fluid with sterile paper points for different periods. Presence of *Candida* was assessed by traditional microbiological culture in blood agar [16, 17] or/and CHROMagar [10, 18, 22, 23]. Identification of clinical isolates of different *Candida* species was also detected by either quantitative real-time PCR [19, 21], random amplified polymorphic DNA [22] or ATB ID 32 [18]. In all cases, the patients had not taken antibiotics, at least 2 months before taking the samples.

These studies analysed 626 patients, 361 with PI (57.7%) and 265 without PI (42.3%), whose mean age varied between 53 and 67 years. Data about gender was available in 5 studies [10, 18, 19, 21, 23], from which 20.3% were women (38 with PI and 33 without PI) and 79.7% (162 with PI and 117 without PI) men. Curiously, two articles from Saudi Arabia did not include female patients [10, 23]. In total, 737 patients wearing dental implants were investigated, 400 (54.3%) with peri-implantitis and 337 (45.7%) without PI (Table 1).

Only one study did not found *Candida* in the sulcular fluid [17]. The rest of the studies recognized a bigger presence of the fungi in the implants with PI (range, 3–76.7%), in contrast to healthy ones (range, 9–50%) [15, 16, 18, 19, 21–23]. Dentate patients also had more *Candida* in their implants than edentulous ones [16, 19]. Fungal colonization was observed only in dental implants with PI in three studies [15, 16, 18].

*C*. *albicans* was the most isolated species, followed by *Candida parapsilosis*, *Candida tropicalis*, *Candida dubliniensis*, *Candida boidinii*, *Candida guilliermondii*, *Candida krusei* and *Candida lusitaniae* (Table 2). Rosenberg et al. [15] did not indicate the species of *Candida*. Most of the authors reported presence of *C*. *albicans*, as well as *C*. *boidinii*, *C*. *dubliniensis*, *C*. *parapsilosis*, *C*. *tropicalis*, *C*. *guilliermondii*, *C*. *krusei* and *C*. *lusitaniae* [10, 21–23]. None of the authors stated if these were pure or polifungal *Candida* biofilms [10, 21–23]. *C*. *albicans* alone was observed in three studies [16–18].

**Table 2:** Microbiological findings of the included studies. Species of *Candida*

Authors and year	***Candida*** presence (%)			Species of ***Candida***
	Total	PI	Healthy	
Rosenberg et al. 1991 [15]	32	32	0	-
Leonhardt et al. 1999 [16]	27	27	0	*C*. *albicans*
Albertini et al. 2015 [18]	3	3	-	*C*. *albicans*
Canullo et al. 2015 [19]	13.5	16.9	15.9%	*C*. *albicans*
Schwarz et al. 2015 [20]	12.9	15.8	10	*C*. *albicans*, *C*. *boidinii*, *C dubliniensis*
Bertone et al. 2016 [21]	51.5	53	50	*C*. *albicans*, *C*. *dubliniensis*, *C*. *parapsilosis*, *C*. *tropicalis*, *C*. *guilliermondii*, *C*. *krusei*, *C*. *lusitaniae*
Alrabiah et al. 2019 [10]	44.5	76.7	12.2	*C*. *albicans*, *C*. *tropicalis*, *C*. *parapsilosis*
Alsahhaf et al. 2019 [22]	43	76.2	9.8	*C*. *albicans*, *C*. *tropicalis*, *C*. *parapsilosis*

*PI* peri-implantitis

### Risk of bias in individual studies

After applying modified NOS assessment, 22.2% of the studies revealed 9 stars, 44.5% of them 8 and 33.3% of them 7 (Table 3). Overall, risk of bias was low.

**Table 3:** Quality assessment of the included studies. NOS tool

Authors, year	Type of study	Newcastle-Ottawa Scale (NOS)		
		Selection	Comparison	Exposure/outcome
Rosenberg et al. 1991 [15]	Case-control	★★	★★	★★★
Leonhardt et al. 1999 [16]	Case-control	★★	★★	★★★
Listgarten et al. 1999 [17]	Cohort	★★★	★★	★★★
Albertini et al. 2015 [18]	Cohort	★★★	★★	★★★
Canullo et al. 2015 [19]	Case-control	★★	★★	★★★
Schwarz et al. 2015 [20]	Case-control	★★★	★★	★★★
Bertone et al. 2016 [21]	Cohort	★★★	★★	★★★
Alrabiah et al. 2019 [10]	Case-control	★★★★	★★	★★★
Alsahhaf et al. 2019 [22]	Case-control	★★★★	★★	★★★

### Clinical and microbiological characteristics of the excluded studies

The six articles excluded in this review analysed the presence of *Candida* in the sulcular fluid surrounding 362 dental implants  [2, 23–27]. In most reports, *C*. *albicans* was isolated [2, 23, 25, 26]; however, *C*. *dubliniensis*, *C*. *glabrata*, *Candida kefyr* and *Candida norvegensis* were also observed [20, 24, 26] (Table 4). The presence of *Candida* was variable, affecting between 10 and 71% of the implants. The study of Peñarrocha et al. [24] was the only one in which no *Candida* was observed in the implants investigated. This absence of *Candida* colonization might be related to the small sample size (20 patients) and to the fact that they look for the presence of *C*. *albicans* and no other species of *Candida*.

**Table 4:** Clinical data and microbiological findings of the excluded studies*

Authors and year (country)	Patients (implants)	Isolation of ***Candida*** from clinical specimens	
		%	Species
Kilic et al. 2014 (Turkey) [23]	37 (37)	71	*C*. *albicans*, *C*. *glabrata*, *C*. *kefyr*, *C*. *norvegensis*
Peñarrocha et al. 2015 (Spain) [24]	20 (43)	0	-
Canullo et al. 2015 (Italy) [25]	40 (80)	15	*C*. *albicans*, *C*. *dubliniensis*
Gomes et al. 2017 (Brazil) [26]	14 (60)	-	*C*. *albicans*, *C*. *dubliniensis*
Mencio et al. 2017 (Italy) [2]	20 (50)	10	*C*. *albicans*
Ju et al. 2019 (South Korea) [27]	92 (92)	13	-

*These articles were excluded due to the absence of any peri-implant diagnostic criteria

Gomes et al. [26] described that the quantity of *Candida* was bigger at 8 months was higher than that observed at 4 months after implant placement. Mencio et al. [2] only observed the presence of *C*. *albicans* in implants with cemented implant-abutment connections but not in those with screwed implant-abutment connections. In addition, Kilic et al. [23] found more *Candida* colonization in patients with bar-retained overdentures (25%) than in those with locator-retained overdentures (19%) (Table 3).

## Discussion

*Candida* is a heterogeneous fungal genus composed by more than 150 species. Although some species of *Candida* coexist as human commensals, they can cause superficial and systemic infections under certain circumstances [28]. Most candidiasis are caused by *C*. *albicans*, but in the recent years other non-*C*. *albicans* species have manifested a pathogenic capacity. Among the most frequently isolated from clinical specimens are *Candida glabrata*, *C*. *parapsilosis* and *C*. *tropicalis* [29].

The pathogenicity of *Candida* responds to a set of virulence factors, including dimorphism, secretion of hydrolytic enzymes (proteases, lipases and haemolysins) and adhesion and biofilm formation on the mucous epithelium and on medical devices [30, 31]. Formation of biofilms is a complex sequential process that depends on the invasive agent and the structure on which it is hosted [32]. Yeast colonization of biotic and abiotic surfaces is the first step in the development of biofilms, followed by cell division and microcolonies generation that contribute to the maturation of a biofilm characterized by the presence of hyphae and yeasts (sessile cells) embedded in an extracellular matrix and, finally, the detachment of some of these cells [33]. The release of planktonic cells into the environment allows them to colonize new surfaces and to develop new foci of candidiasis.

Because the diagnostic criteria of the peri-implant diseases have been in constant change, the diagnosis of peri-implantitis in the included studies have differed from one to another, due to being published over a long period of time, from 1991 to 2020. Yet most of the reviewed studies collected data about bleeding and/or suppuration on probing, probing depth and radiographic bone loss. Moreover, implant mobility and presence of keratinized mucosa was evaluated in three studies [15, 16, 20, 21]. For all these reasons, although it cannot be guaranteed that all the implants studied in this work have been correctly categorized as healthy or diseased, the margin of error could not be very wide [5, 34, 35]. In regards to the risk factor of PI, none of the studies excluded patients with history of periodontitis and two discarded smokers [10, 22]. Still, only one of the nine selected articles did not state whether they found *Candida* or not [17].

According to the included studies of this review, implants with peri-implantitis (range, 3–76.7%) had a higher presence of *Candida* than those without peri-implantitis (range, 9–50%). However, we do not know why prevalence of *Candida* was significantly bigger in the studies with individuals form Saudi Arabia [10, 22]. Since fungal assessment and sample size were similar to other studies [16, 18], we believe these particular results may be related to special geographical and sociocultural factors, such as diet, and the fact that these authors excluded female patients and smokers [10, 22].

Moreover, the fact that 10–71% of implants, regardless of peri-implantitis, showed the presence of this fungus demonstrates that colonization of *Candida* in the peri-implant environment is independent of the disease. Also, because a slightly higher number of patients with bar-retained overdentures had *Candida*, in contrast to locator-retained overdentures, time and hygiene of implants might also be important factors of fungal colonization.

Schwarz et al. [20] were the sole authors to state a direct relationship between *Candida* and other microorganisms. Thereby, from three implants with PI, *C*. *boidinii* was isolated alongside *Mycoplasma salivarum*, *Veillonella parvula*, *Porphyromonas gingivalis*, *Parvimonas micra* and *Tannerella forsythia* in one of them, and in the other two, *C*. *albicans* was also found with *V*. *parvula*, *T*. *forsythia*, *M*. *salivarum*, *P*. gingivalis and *P*. *micra*. On the other hand, *C*. *dubliniensis* was accompanied by *M*. *salivarum*, *V*. *parvula*, *Staphylococcus aureus*, *P*. *micra* and *T*. *forsythia* in one healthy implant. The latter species of *Candida*, *C*. *dubliniensis* has been isolated from patients suffering from different oral pathologies, resembling *C*. *albicans* in many virulence factors including hypha formation and hydrolytic enzyme production [36, 37]. In this context, *Candida* colonization could be linked to the presence of other periodontopathogens, like *T*. *forsythia*, *P*. *micra* or *P*. *gingivalis*. Although the exact role of *Candida* in the beginning of the peri-implant disease is unknown, we believe that this fungus could play an important function in the latter stages of PI, when the bacterial microenvironment is already established, as demonstrated in experimental studies [38].

Knowledge of the involvement of *Candida* infection in disorders of bone remodelling is limited, but it has been described in *Candida* arthritis and osteomyelitis [39, 40], nosological entities described very rarely in the jaws [41, 42]. *Candida* arthritis and osteomyelitis develop by haematogenous invasion, mainly in patients with immunodeficiency, being *C*. *albicans* is usually the most frequently isolated, although *C*. *tropicalis*, *C*. *glabrata* and *C*. *parapsilosis* has also been isolated [40]. As in the latter processes, *Candida* would act as a modifying agent in chronic inflammation around dental implants that activates the bone resorption response.

**Fig. 2:**
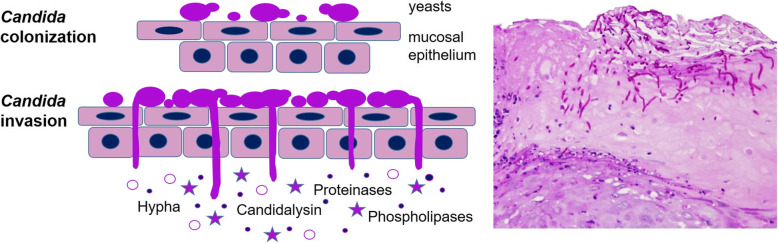
*Candida albicans* virulence factors. Mechanisms by which *C*. *albicans* promotes colonization and invasion of mucosal cells

Anaerobiosis, as occurs in peri-implant pockets, can promote the virulence of *C*. *albicans*, increasing the activity of secreted aspartyl proteinases (Sap) [43]. These Sap proteins are associated to *Candida* adherence, tissue damage and modulation of immune response, maintaining inflammatory stimuli that attract other periodontopathogens [44, 45]. This role of Saps is important because the ability to form thick biofilms is easier for *C*. *albicans*, both under aerobic and anaerobic conditions, whereas for the rest of the species of *Candida*, growth is much greater only under aerobic conditions [46]. *C*. *albicans* hyphae secrete candidalysin, a 31-amino-acid peptide toxin that damages the epithelial cells and has an immunomodulatory capacity by binding to epidermal growth factor receptor (ErbB1 or Her1) [47]. Yeast and hyphal morphologies are present during asymptomatic *C*. *albicans* colonization of human mucosal surfaces. However, hypha formation can lead to candidalysin secretion, tissue damage and immune modulation (Fig. 2). Furthermore, *C*. *albicans* 95-kDa metallopeptidase, localized in cell wall, owns the capacity to destroy different elements of the peri-implant soft and hard tissues like type I collagen (connective tissue, alveolar bone and cement), type IV collagen (basement membrane of the mucosa), fibronectin (periodontal ligament) and laminin (basement membrane of the mucosa and cement) [48–50].

Interestingly, one study [22] pointed that patients with dental implants in which *Candida* was isolated, also had a higher presence of these fungi at the buccal, lingual and palatal mucosa. Although this study did not differentiate between implants with and without PI, it evidences the existence of an oral reservoir for *Candida* that facilitates the entry of this fungus into the peri-implant sulcus.

This systematic review has limitations. First, only nine studies analysed the presence of *Candida* in patients with peri-implantitis including very heterogeneous samples (range, 20–126 studied specimens). Second, few studies reported the number of *Candida* colony-forming units (CFU), which is a fundamental data for the mycological analysis [9, 51]. Alrabiah et al. [10] showed significant differences in the quantity of *Candida* between specimens from patients suffering PI (3147.54 CFU/mL) and from patients without PI (496.68 ± 100.2 CFU/mL). These findings are similar to those found by Alsahhaf et al. [22] (2316.26 vs 177.6 CFU/ml). Third, it was impossible to analyse the differences of *Candida* in patients with PI, regarding the type, composition, design and surface of the implants, because only Leonhardt et al. [16], said that their implants were Nobel Biocare AB (Gothenburg, Sweden). We are convinced of the importance of studying the implant characteristics, since they can be strongly related to colonization and infection, according to how they allow *Candida* adherence. There is a strong association between implant properties and microbial adhesion, titanium being one of the most resistant to *Candida* colonization and biofilm development [8]. Moreover, there is a considerable heterogeneity of the selected studies regarding the microbial methods and study design then results obtained from different microbiological methods did not allow for a direct comparison.

## Conclusions

In summary, *Candida* is a common inhabitant of the peri-implant sulcular sulcus microbial environment, both in healthy implants and in those from people suffering from PI. However, *Candida* presence is more common in peri-implantitis. This presence and the *Candida* concentrations in the peri-implant tissue can be related to the lapse of time dental implants have been in the oral cavity. In addition, the quantity of *Candida* in the sulcular fluid surrounding the implants might rely on the presence of other periodontopathogens, such as *V*. *parvula*, *T*. *forsythia*, *M*. *salivarum*, *P*. *gingivalis* or *P*. *micra*. Although, *C*. *albicans*, *C*. *parapsilosis* and *C*. *tropicalis* are the most common species in peri-implantitis, others have also been identified, such as *C*. *dubliniensis*, *C*. *boidinii*, *C*. *guilliermondii*, *C*. *krusei* and *C*. *lusitaniae*, but in a very low frequency. Better-designed studies are needed, with larger patient samples, to unravel whether there is a relevant role for *Candida* in the etiopathogenesis of peri-implantitis and dental implant failure.

## Data Availability

All data generated or analysed during this study are included in this published article [and its supplementary information files].

## References

[1] Karoussis IK, Salvi GE, Heitz-Mayfield LJ, Brägger U, Hämmerle CH, Lang NP (2003). Long-term implant prognosis in patients with and without a history of chronic periodontitis: a 10-year prospective cohort study of the ITI® Dental Implant System. Clin Oral Implants Res..

[2] Mencio F, De Angelis F, Papi P, Rosella D, Pompa G, Di Carlo S (2017). A randomized clinical trial about presence of pathogenic microflora and risk of peri-implantitis: comparison of two different types of implant-abutment connections. Eur Rev Med Pharmacol Sci..

[3] Schwarz F, Derks J, Monje A, Wang HL (2018). Peri-implantitis. J Clin Periodontol..

[4] Berglundh T, Zitzmann NU, Donati M (2011). Are peri-implantitis lesions different from periodontitis lesions?. J Clin Paeriodontol..

[5] Persson GR, Renvert S (2014). Cluster of bacteria associated with peri-implantitis. Clin Implant Dent Rel Res..

[6] Bürgers R, Hahnel S, Reichert TE, Rosentritt M, Behr M, Gerlach T, Handel G, Gosau M (2010). Adhesion of *Candida albicans* to various dental implant surfaces and the influence of salivary pellicle proteins. Acta Biomat..

[7] Cobo F, Rodríguez-Granger J, Sampedro A, Aliaga-Martínez L, Navarro-Marí JM (2017). Candida prosthetic joint infection. A review of treatment methods. J Bone Joint Inf..

[8] De-la-Pinta I, Cobos M, Ibarretxe J, Montoya E, Eraso E, Guraya T, Quindós G (2019). Effect of biomaterials hydrophobicity and roughness on biofilm development. J Mater Sci Mater Med..

[9] Aguirre-Urizar JM (2002). Candidiasis orales. Rev Iberoam Micol..

[10] Alrabiah M, Alshagroud RS, Alsahhaf A, Almojaly SA, Abduljabbar T, Javed F (2019). Presence of Candida species in the subgingival oral biofilm of patients with peri-implantitis. Clin Implants Dent Relat Res..

[11] Urzúa B, Hermosilla G, Gamonal J, Morales-Bozo I, Canals M, Barahona (2008). Yeast diversity in the oral microbiota of subjects with periodontitis: *Candida albicans* and *Candida dubliniensis* colonize the periodontal pockets. Sabouraudia..

[12] De la Torre J, Quindós G, Marcos-Arias C, Marichalar-Mendia X, Gainza ML, Eraso E (2018). Oral Candida colonization in patients with chronic periodontitis. Is there any relationship?. Rev Iberoam Micol..

[13] Moher D, Liberati A, Tetzlaff J, Altman DG (2009). Preferred reporting items for systematic reviews and meta-analyses: the PRISMA statement. An Int Med..

[14] Wells GA, Tugwell P, O’Connell D, Welch V, Peterson J, Shea B (2015). The Newcastle-Ottawa Scale (NOS) for assessing the quality of nonrandomized studies in meta-analyses.

[15] Rosenberg ES, Torosian JP, Slots J (1991). Microbial differences in 2 clinically distinct types of failures of osseointegrated implants. Clin Oral Implants Res..

[16] Leonhardt Å, Renvert S, Dahlén G (1999). Microbial findings at failing implants. Clin Oral Implants Res..

[17] Listgarten MA, Lai CH (1999). Comparative microbiological characteristics of failing implants and periodontally diseased teeth. J Periodontol..

[18] Albertini M, López-Cerero L, O'Sullivan MG, Chereguini CF, Ballesta S, Ríos (2015). Assessment of periodontal and opportunistic flora in patients with peri-implantitis. Clin Oral Implants Res..

[19] Canullo L, Peñarrocha-Oltra D, Covani U, Rossetti PHO (2015). Microbiologic and clinical findings of implants in healthy condition and with peri-implantitis. Int J Oral Maxillofac Implants..

[20] Canullo L, Penarrocha-Oltra D, Soldini C, Mazzocco F, Penarrocha M, Covani U (2015). Microbiological assessment of the implant-abutment interface in different connections: cross-sectional study after 5 years of functional loading. Clin Oral Implants Res..

[21] Schwarz F, Becker K, Rahn S, Hegewald A, Pfeffer K, Henrich B (2015). Real-time PCR analysis of fungal organisms and bacterial species at peri-implantitis sites. Int J Implant Dent..

[22] Bertone AM, Rosa AC, Nastri N, Santillán HD, Ariza Y, Iovannitti CA (2016). Genetic-relatedness of peri-implants and buccal *Candida albicans* isolates determined by RAPD-PCR. Acta Odonto Lat..

[23] Alsahhaf A, Al-Aali KA, Alshagroud RS, Alshiddi IF, Alrahlah A, Abduljabbar T (2019). Comparison of yeasts species in the subgingival oral biofilm of individuals with type 2 diabetes and peri-implantitis and individuals with peri-implantitis without diabetes. J Periodontol..

[24] Kilic K, Koc AN, Tekinsen FF, Yildiz P, Kilic D, Zararsiz G, Kilic E (2014). Assessment of Candida species colonization and denture-related stomatitis in bar-and locator-retained overdentures. J Implantol..

[25] Peñarrocha-Oltra D, Rossetti PH, Covani U, Galluccio F, Canullo L (2015). Microbial leakage at the implant-abutment connection due to implant insertion maneuvers: cross-sectional study 5 years postloading in healthy patients. J Oral Implantol..

[26] Gomes JA, Sartori IA, Able FB, de Oliveira Silva TS, do Nascimento C (2017). Microbiological and clinical outcomes of fixed complete-arch mandibular prostheses supported by immediate implants in individuals with history of chronic periodontitis. Clin Oral Implants Res..

[27] Ju HM, Ahn YW, Jeong SH, Jeon HM, Kim KH, Song BS, Ok SM (2019). Characteristics of patients who perceive dental treatment as a cause of oral mucosal lesions. J Oral Sci..

[28] Araújo D, Henriques M, Silva S (2017). Portrait of Candida species biofilm regulatory network genes. Trend Microbiol..

[29] Quindós G, Marcos-Arias C, San-Millán R, Mateo E, Eraso E (2018). The continuous changes in the aetiology and epidemiology of invasive candidiasis: from familiar *Candida albicans* to multiresistant *Candida auris*. Int Microbiol..

[30] Berman J, Sudbery PE (2002). Candida albicans: a molecular revolution built on lessons from budding yeast. Nat Rev Genet..

[31] Mayer FL, Wilson D, Hube B (2013). Candida albicans pathogenicity mechanisms. Virulence..

[32] Nobile CJ, Fox EP, Nett JE, Sorrells TR, Mitrovich QM, Hernday AD, Tuch BB, Andes DR, Johnson AD (2012). A recently evolved transcriptional network controls biofilm development in *Candida albicans*. Cell..

[33] Finkel JS, Mitchell AP (2012). Genetic control of *Candida albicans* biofilm development. Nat Rev Microbiol..

[34] Kumar PS, Mason MR, Brooker MR, O'Brien K (2012). Pyrosequencing reveals unique microbial signatures associated with healthy and failing dental implants. J Clin Periodontol..

[35] Carcuac O, Berglundh T (2014). Composition of human peri-implantitis and periodontitis lesions. J Dent Res..

[36] Gutiérrez J, Morales P, González MA, Quindós G (2002). *Candida dubliniensis*, a new fungal pathogen. J Basic Microbiol..

[37] Sahand IH, Maza JL, Eraso E, Montejo M, Moragues MD, Aguirre JM, Quindós G, Pontón J (2009). Evaluation of CHROM-Pal medium for the isolation and direct identification of *Candida dubliniensis* in primary cultures from the oral cavity. J Med Microbiol.

[38] Cavalcanti YW, Wilson M, Lewis M, Del-Bel-Cury AA, da Silva WJ, Williams DW (2016). Modulation of *Candida albicans* virulence by bacterial biofilms on titanium surfaces. Biofoul..

[39] Slenker AK, Keith SW, Horn DL (2012). Two hundred and eleven cases of Candida osteomyelitis: 17 case reports and a review of the literature. Diagn Microbiol Infect Dis..

[40] Gamaletsou MN, Rammaert B, Bueno MA, Sipsas NV, Moriyama B, Kontoyiannis D (2016). Candida arthritis: analysis of 112 pediatric and adult cases. Open Forum Infect Dis.

[41] Attie MD, Anderson IA, Portnof J (2018). Mandibular osteomyelitis associated with *Candida albicans* in marijuana and heroin abusers. Ann Maxillofac Surg..

[42] Kaushal D, Sharma A, Kesarwani A, Kalita JM (2019). Chronic Candida osteomyelitis of hard palate and nose: a diagnostic quandary. Med Mycol Case Rep..

[43] Rosa EAR, Rached RN, Ignácio SA, Rosa RT, da Silva WJ, Yau JYY (2008). Phenotypic evaluation of the effect of anaerobiosis on some virulence attributes of *Candida albicans*. J Med Microbiol..

[44] Naglik JR, Challacombe SJ, Hube B (2003). *Candida albicans* secreted aspartyl proteinases in virulence and pathogenesis. Microbiol Mol Biol Rev..

[45] Sardi JCO, Scorzoni L, Bernardi T, Fusco-Almeida AM, Giannini MM (2008). Candida species: current epidemiology, pathogenicity, biofilm formation, natural antifungal products and new therapeutic options. J Med Microbiol..

[46] Thein ZM, Samaranayake YH, Samaranayake LP (2007). In vitro biofilm formation of *Candida albicans* and non-albicans Candida species under dynamic and anaerobic conditions. Arch Oral Biol..

[47] Naglik JR, Gaffen SL, Hube B (2019). Candidalysin: Discovery and function in *Candida albicans* infections. Curr Opin Microbiol..

[48] Rodier MH, El Moudni B, Kauffmann-Lacroix C, Daniault G, Jacquemin JL (1999). A *Candida albicans* metallopeptidase degrades constitutive proteins of extracellular matrix. FEMS Microbiol Let..

[49] Embery G, Waddington RJ, Hall RC, Last KS (2000). Connective tissue elements as diagnostic aids in periodontology. Periodontol 2000.

[50] Canabarro A, Valle C, Farias MR, Santos FB, Lazera M, Wanke B (2013). Association of subgingival colonization of *Candida albicans* and other yeasts with severity of chronic periodontitis. J Periodontol Res..

[51] Tooyama H, Matsumoto T, Hayashi K, Kurashina K, Kurita H, Uchida M, Kasuga E, Honda T (2015). Candida concentrations determined following concentrated oral rinse culture reflect clinical oral signs. BMC Oral Health..

